# Effect of dexmedetomidine on pulmonary function in obese patients undergoing laparoscopic surgery

**DOI:** 10.1186/s13741-024-00396-6

**Published:** 2024-05-16

**Authors:** Huan Chen, Xin Wang, Yawen Zhang, Wei Liu, Changhao Zhou, Deli Zheng

**Affiliations:** Department of Anesthesiology, The First Hospital of Hebei Medical University, Shijiazhuang, 050000 Hebei China

**Keywords:** Dexmedetomidine, Obesity, Laparoscopy, Lung protection, General anesthesia, Inflammatory markers

## Abstract

**Objective:**

This research aimed to ascertain the effect of dexmedetomidine on pulmonary function in obese patients undergoing laparoscopic surgery.

**Methods:**

Obese patients undergoing laparoscopic surgery under general anesthesia were separated into the control group (group C) and the dexmedetomidine group (group D) (*n* = 30). Patients in group D were infused with dexmedetomidine (1 μg/kg) intravenously for 10 min and then at a rate of 0.5 mg/kg h until 30 min before the end of the surgery, and those in group C were infused with an equal volume of saline. The surgery time points were divided into: before anesthesia induction (T0), 5 min after intubation (T1), 30 min after pneumoperitoneum (T2), 10 min after pneumoperitoneum release (T3), at the time of extubation (T4), 3 min after extubation (T5), and 24 h after surgery (T6). Arterial blood was collected for blood gas analysis to record arterial partial pressure of oxygen (PaO_2_) and arterial partial pressure of carbon dioxide (PaCO_2_). Dynamic lung compliance (Cdyn), oxygenation index (OI), alveolar-arterial oxygen partial pressure difference (A-aDO_2_), and respiratory index (RI) were calculated. The time of surgery, anesthesia, CO_2_ pneumoperitoneum, eye-opening, and time from the end of surgery to extubation were recorded. Plasma IL-8 and IL-10 levels were measured from T0 to T6.

**Results:**

The time of surgery, anesthesia, CO_2_ pneumoperitoneum, eye-opening, and time from the end of surgery to extubation in group D were not statistically significant when compared with those in group C. Versus at the T1 time point, A-aDO_2_ and RI were higher and Cdyn and OI were lower in both groups at T2 and T3 time points. Versus group C, group D had higher Cdyn and OI and lower A-aDO_2_ and RI at T2 and T3 time points. Versus at the T0 time point, at each time point from T1 to T6, IL-8 and IL-10 levels were higher in both groups. Versus group C, group D had lower IL-8 and higher IL-10 levels at each time point from T1 to T6.

**Conclusion:**

In obese patients undergoing laparoscopic surgery under general anesthesia, the use of dexmedetomidine can improve the lung compliance and OI of the patients, inhibit the inflammatory response of the lungs of the patients and thus have a certain protective effect on the lung function.

## Introduction

Obesity is a public health epidemic that is projected to increase in the coming years. Morbidly obese patients and obese patients are not only at significantly higher risk for any complication but also at higher odds for worse medical and economic outcomes after surgery (Hussein, et al. 2022). Obesity presents challenges to both physicians and patients and it also has a negative impact on health conditions (National Task Force on the, P. and O 2002). The optimization of mechanical ventilation for critically ill patients with obesity is one of the greatest challenges (Kacmarek et al. 2021). Moreover, managing ventilation and oxygenation during laparoscopic surgery in severely obese patients experiencing weight loss surgery shows many challenges (Dion et al. 2014).

Dexmedetomidine is regarded as an attractive adjunct to general and regional anesthesia for a variety of procedures (Mahmoud and Mason 2015). It is also reported that dexmedetomidine has the ability to protect cells and organs because of its direct cellular impact. More specifically, dexmedetomidine can raise vagal nerve tone that partially contributed to its anti-inflammatory and lung-protective effects (Li et al. 2022). Dexmedetomidine may improve intrapulmonary shunt and arterial oxygenation during one-lung ventilation (OLV) in adults receiving thoracic surgery (Huang et al. 2017; Wang et al. 2022). The improved oxygenation results in less demand of volatile anesthetic (isoflurane) agents in patients receiving dexmedetomidine (Asri et al. 2020). Bai et al. have found that perioperative dexmedetomidine administration can alleviate OLV-induced inflammation, improve pulmonary oxygenation, and may help reduce postoperative complications and better prognosis (Bai et al. 2021). Wang et al. have supported that a loading dose of 0.25 μg/kg and a maintenance dose of 0.3 μg/kg/h infusion of dexmedetomidine can improve dynamic lung compliance in patients accepting desflurane during general anesthesia (Wang et al. 2023). Dexmedetomidine improves oxygenation in patients with OLV, shortens the length of hospital stay, and reduces the incidence of postoperative pulmonary complications, which may be associated with improvements in anti-inflammatory effects, lung compliance, as well as regulation of oxidative stress reactions (Yang et al. 2023). The pharmacodynamics and pharmacokinetics of dexmedetomidine vary significantly in morbidly obese patients in contrast with patients with normal weight. The levels of sedation are markedly deeper, and oxygen saturation is lower in morbidly obese patients, possibly resulting from higher plasma concentration after a 1.0-µg/kg infusion (Xu et al. 2017). In a previous study, it demonstrated that an infusion of 90-min dexmedetomidine can lead to moderate improvement in oxygenation and lung mechanics in morbidly obese patients with restrictive lung disorders (Hasanin et al. 2018). From the above studies, we can postulate that dexmedetomidine has anti-inflammatory or cell protective effect. In our paper, the study focused on the effects of dexmedetomidine in obese patients undergoing laparoscopic surgery under general anesthesia.

## Materials and methods

### Ethics statement

The study was under the approval of the Ethic Committee of The First Hospital of Hebei Medical University (approval number: 2023-00036), and the patients gave written informed consent.

### Study subjects

Obese patients undergoing elective laparoscopic surgery under general anesthesia were selected for our experiment. Inclusion criteria: age 18–65 years, ASA class II or III, 30 kg/m^2^ ≤ body mass index (BMI) < 40 kg/m^2^, and unlimited gender. Exclusion criteria: patients with anticipated difficulty in mask ventilation and obstructive sleep apnea syndrome; those with severe cardiopulmonary, hepatic, and renal diseases; those with alcoholism, drug abuse, and long-term use of sedative and analgesic drugs; those with psychiatric disorders and those who were unable to cooperate; those with a third-degree atrioventricular block with an entry heart rate (HR) < 50 beats/min. On the basis of the random number table method, patients were randomly separated into 2 groups (*n* = 30): the control group (group C) and the dexmedetomidine group (group D). The trial participants, anesthetists, staff collecting data, and laboratory staff were blinded to the group assignments.

### Test methods

Patients underwent routine preoperative fasting. Upon entering the operating room, an intravenous cannula was sited in the upper limb, and pulse oximetry, electrocardiogram, and noninvasive blood pressure were tested. The arterial pressure was measured by direct puncture of the radial artery under local anesthesia with lidocaine. Patients in group D were given dexmedetomidine hydrochloride (1 μg/kg lean body weight (LBW) (calculated as (9270 × body weight)/(6680 + 216 × body weight/height^2^) for males and (9270 × body weight)/(8780 + 244 × body weight/height^2^) for females) 10 min before anesthesia induction, which was subsequently infused at a rate of 0.5 mg/kg h (LBW) until 30 min before the end of the operation, and patients in group C were infused with an equal volume of saline in the same way. Induction of anesthesia: midazolam 0.05–0.1 mg/kg (LBW), sufentanil 0.5–1.0 μg/kg (LBW), propofol 1–2 mg/kg (LBW), and rocuronium bromide 0.6–0.9 mg/kg (LBW) were intravenously injected for 2 min, and then tracheal tube was inserted, to make sure that tracheal tube was correctly positioned, and then connecting to the anesthesia machine to carry out mechanical ventilation. We adopted a volume-controlled mode of ventilation, maintaining a low tidal volume of 6–8 ml/kg LBW, a ventilation frequency of 12–14 times/min, an inhalation to exhalation ratio (I: E) of 1: 2, and a positive end-expiratory pressure (PEEP) of 5 cmH_2_O (1 cmH_2_O = 0.098 kPa). The pressure of end-tidal carbon dioxide (ETCO_2_) was maintained between 35 and 45 mmHg (1 mmHg = 0.133 kPa) by adjusting the respiratory rate. Anesthesia was maintained with propofol 6–8 mg/kg h (LBW), remifentanil 0.1–0.5 μg/kg·min (LBW), and intermittent bolus injection of rocuronium for maintaining muscle relaxation. Ten minutes after general anesthesia was stabilized, CO_2_ was injected into the abdominal cavity (pneumoperitoneum pressure 12 mmHg). Intraoperative fluctuations in mean arterial pressure (MAP) were maintained at no more than 20% of the basal value, and the bispectral index (BIS) value was between 40 and 60. If intraoperative hypotension or bradycardia occurred in the patient, the appropriate cardiovascular active drugs (phenylephrine or atropine) were administered.

The patients were transported to the PACU after the operation. All patients were extubated after receiving an intravenous sugammadex injection at 2 mg/kg of LBW. All patients were evaluated by the anesthesiologist in the PACU and returned to the ward by the nurse anesthetist after meeting the PACU discharge criteria.

### Observation indicators

The surgical time points were divided into: before anesthesia induction (T0), 5 min after intubation (T1), 30 min after pneumoperitoneum (T2), 10 min after pneumoperitoneum release (T3), at the time of extubation (T4), 3 min after extubation (T5), and 24 h after surgery (T6). At four time points T1, T2, T3, and T4, radial artery blood was taken for blood gas analysis, and arterial partial pressure of oxygen (PaO_2_), arterial partial pressure of carbon dioxide (PaCO_2_) values were recorded. Dynamic lung compliance (Cdyn) (calculated as = tidal volume/(peak inspiratory pressure—positive end-expiratory pressure), oxygenation index (OI) (calculated as PaO_2_/FiO_2_), alveolar-arterial oxygen partial pressure difference (A-aDO_2_) (FiO_2_[PB − PH_2_O] − [PaCO_2_/R]) − PaO_2_, PB was the barometric pressure (760 mmHg at sea level); and PH_2_O was the water vapor pressure (47 mmHg when air is fully saturated at 37 ℃)), and respiratory index (RI) (calculated as P(A-a)O_2_/PaO_2_) was calculated based on the formula. The surgery time, anesthesia time, CO_2_ pneumoperitoneum time, eye-opening time, and time from the end of surgery to extubation were recorded. Radial artery blood was taken from T0 to T6, let stand for 30 min, and centrifuged at 3000 r/min for 10 min, and the supernatant was taken and stored at − 70 ℃. Plasma IL-8 and IL-10 concentrations were measured by enzyme-linked immunosorbent assay. The reagent kit was purchased from ACROBiosystems (Beijing, China), and the operating procedures strictly followed the instructions of the reagent kit.

### Statistics

The sample size was determined to study a clinically relevant difference between the two groups with regard to the primary outcome variable. The primary outcome was dynamic lung compliance (Cdyn). Preliminary estimates put the number of cases in each group at 25 (with a two-sided 5% significance level and 80% statistical power). Considering the influence of shedding and elimination (20%), the overall sample size was set as 60 cases, 30 cases in the dexmedetomidine group (group D), and 30 cases in the control group (group C).

The Statistical Package for Social Science (SPSS) software, version 26 for Microsoft Windows (SPSS Inc, Chicago, IL, USA), was used for the data analysis. Measurement data were tested for normality using the Shapiro–Wilk test and were depicted as mean ± standard deviation. The *t*-test was utilized for inter-group comparison. A repeated-measures analysis of variance and the Bonferroni post-hoc pairwise comparison tests were used to assess the changes at the different time points. Numeration data were expressed as number (*n*) and compared by implementing the *χ*^2^ test. Two-sided *P* < 0.05 was considered a statistically significant difference.

## Results

### General data

No statistically significant difference was found in terms of age, gender, BMI, and ASA class between the two groups, which was comparable (*P* > 0.05; Table 1).

**Table 1:** General data between the two groups

	Group C (*n* = 30)	Group D (*n* = 30)
Age (years)	36.57 ± 10.53	37.97 ± 10.85
Gender (cases)		
Female	18	16
Male	12	14
Body mass index (kg/m^2^)	35.15 ± 2.15	34.58 ± 2.74
ASA class (cases)		
II	17	14
III	13	16

### Clinical indicators

When comparing the time of surgery, anesthesia, CO_2_ pneumoperitoneum, eye-opening, and time from the end of surgery to extubation in group D with that in group C, there was no statistical significance (*P* > 0.05; Table 2).

**Table 2:** Clinical indicators between the two groups

Indicator	Group C (*n* = 30)	Group D (*n* = 30)
Surgery time (min)	105.79 ± 18.30	107.45 ± 18.22
Anesthesia time (min)	144.99 ± 20.87	145.64 ± 18.11
CO_2_ pneumoperitoneum time (min)	86.23 ± 10.65	89.61 ± 10.61
Eye-opening time (min)	7.62 ± 2.66	7.14 ± 2.11
Time from the end of surgery to extubation (min)	12.78 ± 0.56	12.88 ± 0.35

### Various indicators of lung function

**Fig. 1:**
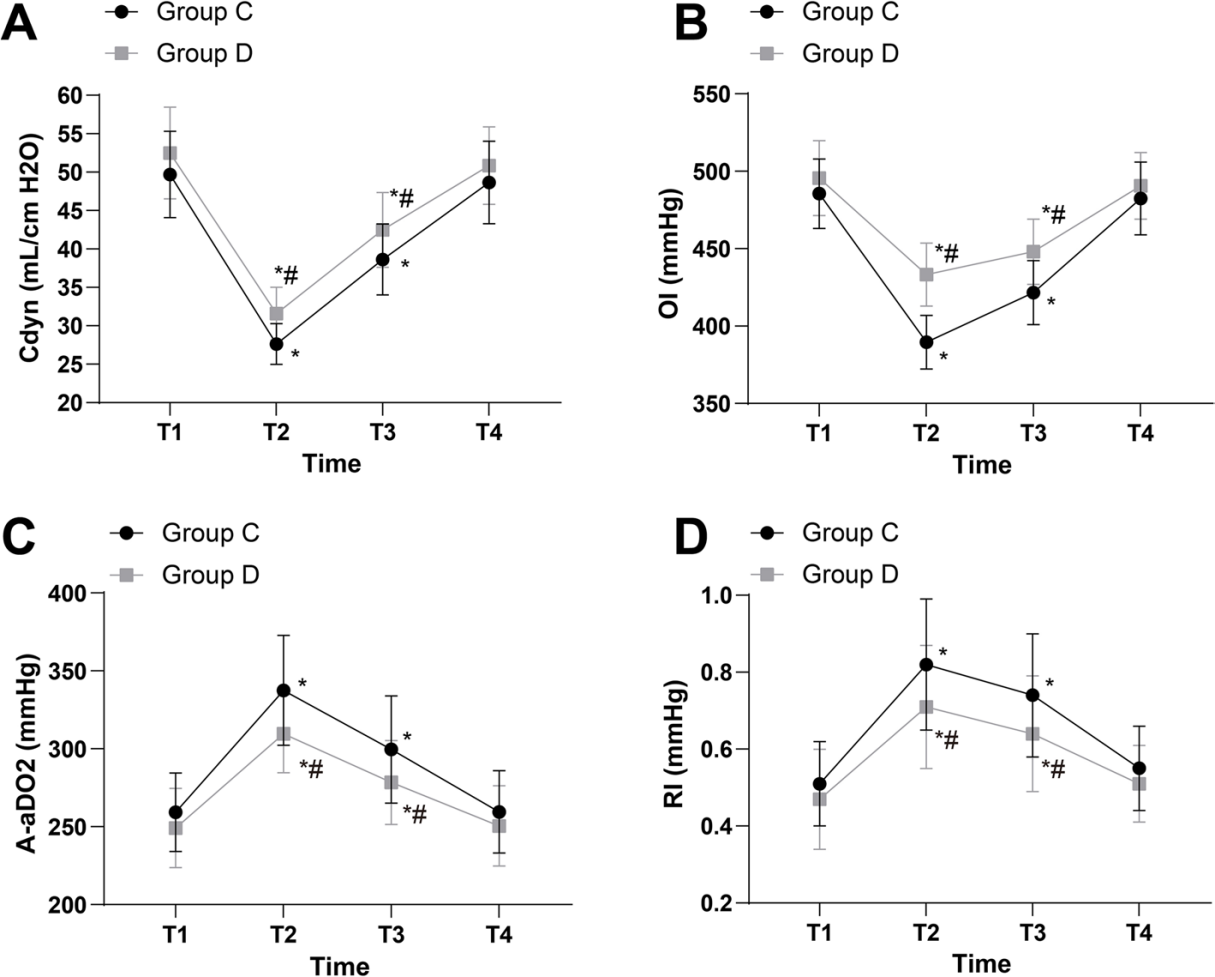
Various indicators of lung function. **A** Comparisons of Cdyn in patients of two groups. **B** Comparisons of OI in patients of two groups. **C** Comparisons of A-aDO_2_ in patients of two groups. **D** Comparisons of RI in patients of two groups. * *P* < 0.05 vs T0; # *P* < 0.05 vs group C

Versus at the T1 time point, group D and group C had reduced Cdyn at the T2 and T3 time points (*P* < 0.05). Versus group C, group D possessed higher Cdyn at the T2 and T3 time points (*P* < 0.05) (Fig. 1A).

Versus at the T1 time point, A-aDO_2_ and RI were higher and OI were lower in group D and group C at the T2 and T3 time points (*P* < 0.05). Versus group C, OI were higher and A-aDO_2_ and RI were lower in group D at the T2 and T3 time points (*P* < 0.05) (Fig. 1B–D).

### Inflammatory markers

Versus at the T0 time point, IL-8 and IL-10 levels in groups C and D were raised at each time point from T1 to T6 (*P* < 0.05). Versus group C, group D possessed lower IL-8 levels and higher IL-10 levels at each time point from T1 to T6 (*P* < 0.05) (Table 3).

**Table 3:** Inflammatory factors between the two groups

	IL-8 (pg/mL)		IL-10 (pg/mL)	
	Group C	Group D	Group C	Group D
T0	39.14 ± 5.51	39.44 ± 5.65	19.61 ± 2.51	19.21 ± 2.72
T1	47.51 ± 5.51*	42.58 ± 5.71*#	22.71 ± 2.94*	25.61 ± 2.69*#
T2	49.51 ± 6.54*	45.89 ± 6.97*#	29.46 ± 2.48*	33.74 ± 2.81*#
T3	57.74 ± 7.54*	50.54 ± 6.94*#	36.44 ± 2.71*	39.67 ± 2.46*#
T4	89.54 ± 17.55*	63.51 ± 15.63*#	56.65 ± 4.85*	61.94 ± 5.86*#
T5	68.61 ± 12.59*	44.64 ± 10.48*#	41.34 ± 5.19*	54.51 ± 4.61*#
T6	56.51 ± 7.64*	52.61 ± 6.87*#	33.51 ± 2.61*	46.02 ± 2.69*#

^*^ *P* < 0.05 vs T0

^#^ *P* < 0.05 vs group C

## Discussion

Obesity can hinder laparoscopic procedures during laparoscopic surgery (Park et al. 2022). In patients who are obese and morbidly obese, the complexity of laparoscopic surgery grows in a substantial manner (Albayati et al. 2023). The increase in oxygen demand, combined with anatomical and physiological changes related to excessive adipose tissue, makes the maintenance of oxygenation a great challenge in the induction, maintenance, as well as recovery process of general anesthesia (Ortiz et al. 2015). Therefore, it is of great clinical value to investigate the effects of dexmedetomidine in obese patients undergoing laparoscopic surgery. The results of this article revealed that the use of dexmedetomidine can improve lung compliance and OI of obese patients undergoing laparoscopic surgery under general anesthesia, inhibit the inflammatory response of the lungs of the patients, and thus have a certain protective effect on lung function.

As previously reported, dexmedetomidine administration may be effective for patients susceptible to ventricular arrhythmia development during robot-assisted laparoscopic prostatectomy (Kim et al. 2016). Patients receiving dexmedetomidine experience less pain and possess lower analgesic requirements. Dexmedetomidine provides more comfort during the procedure for the patient and clinician (Barends, et al. 2017). In our paper, it was found that there was no statistical significance in terms of time of surgery, anesthesia, CO_2_ pneumoperitoneum, eye-opening, and time from the end of surgery to extubation in group D versus in group C, which was similar to the findings in previous research. No differences were found in ASA grade, time of endotracheal extubation, and recovery time for orientation between the dexmedetomidine and control groups (Zhu et al. 2022).

Lung compliance refers to the response of lung volume to changes in unit pressure and is a sensitive marker of lung injury and lung ventilation. High lung compliance can to some extent reflect lower respiratory tract reactions. It is vital to improve lung compliance in general anesthesia patients, decrease respiratory work, and promote gas exchange (Wang et al. 2023). A previous study has shown significant increases with Cdyn after OLV in dexmedetomidine 0.5, dexmedetomidine 1, and dexmedetomidine 2 groups (Xu et al. 2020). In our study, we found that versus at the T1 time point, both groups had elevated reduced Cdyn at T2 and T3 time points. Versus group C, group D possessed higher Cdyn at T2 and T3 time points. As reported, the inhibition of adverse reactions to tracheal intubation mediated by dexmedetomidine and the stability of respiratory status during surgery may be the reasons for high Cdyn after dexmedetomidine administration (Wang et al. 2023).

OI reflects the gas exchange function of the lungs; the larger OI presents better lung ventilation function. The physiological impacts of single-lung ventilation often result in oxygenation dysfunction (Wang et al. 2023). Subsequently, we compared gas exchange function indicators between the two groups and found that versus at the T1 time point, A-aDO_2_ and RI were higher and OI was lower in both groups at T2 and T3 time points. Versus group C, OI was higher and A-aDO_2_ and RI were lower in group D at T2 and T3 time points. These trends were consistent with previous research results. The patients who had received dexmedetomidine and a lung-protective ventilation strategy had higher PaO_2_ and OI. Dexmedetomidine combined with a pulmonary protective ventilation strategy can reduce perioperative lung injury in patients undergoing surgery by repressing inflammatory responses and oxidative stress to promote lung function and decrease adverse effects of the surgery (Gong et al. 2020).

Evidence has shown (Sanders and Maze 2007) that dexmedetomidine has superior anti-inflammatory effects, which can enhance macrophage action, anti-apoptosis, does not affect the chemotactic phagocytosis of neutrophils, does not affect the production of superoxide anion, and is also able to maintain the activity of natural killer cells and limit the reduction of the inflammatory response caused by endotoxin after surgery and general anesthesia. Other researchers and scholars (Yang et al. 2008) have also demonstrated through animal experiments that dexmedetomidine inhibits the release of inflammatory factors in ventilatory lung injury. IL-8 is considered to be a specific cytokine for inflammatory response and tissue injury in the lungs and can reflect the degree of lung injury. IL-10, as an anti-inflammatory factor, can cause mononuclear macrophages to reduce the synthesis and release of inflammatory mediators and inhibit the intensity of the inflammatory response. This study was to investigate the lung-protective effect of dexmedetomidine in laparoscopic surgery in obese patients. Therefore, we measured the levels of IL-8 and IL-10. In our paper, we found that versus at the T0 time point, at each time point from T1 to T6, IL-8 and IL-10 levels in groups C and D were raised. Versus group C, group D possessed lower IL-8 levels and higher IL-10 levels at each time point from T1 to T6.

In summary, this research demonstrates that in obese patients undergoing laparoscopic surgery under general anesthesia, the use of dexmedetomidine can improve the lung compliance and OI of the patients, inhibit the inflammatory response of the lungs of the patients, and thus have a certain protective effect on the lung function. This study lays a foundation to study the clinical value of dexmedetomidine in obese patients. The application of the present findings to clinical practice may reduce the inflammatory response in obese patients undergoing laparoscopic surgery under general anesthesia, provide a new avenue for perioperative lung protection, and promote patient recovery. Nevertheless, our study has limitations. Due to the immature design of this study, we did not focus on observing the patient’s functional performance (length of hospital stay, time to resume normal activities, etc.) or subjective well-being (pain score, anxiety level), which resulted in incomplete data. Meanwhile, our study is based on limited clinical data, and further larger studies powered for patient-centered outcomes may be worthwhile to further confirm our findings.

## Data Availability

No datasets were generated or analysed during the current study.
